# Novel Barite Chimneys at the Loki's Castle Vent Field Shed Light on Key Factors Shaping Microbial Communities and Functions in Hydrothermal Systems

**DOI:** 10.3389/fmicb.2015.01510

**Published:** 2016-01-07

**Authors:** Ida H. Steen, Håkon Dahle, Runar Stokke, Irene Roalkvam, Frida-Lise Daae, Hans Tore Rapp, Rolf B. Pedersen, Ingunn H. Thorseth

**Affiliations:** ^1^Centre for Geobiology, University of BergenBergen, Norway; ^2^Department of Biology, University of BergenBergen, Norway; ^3^Department of Earth Science, University of BergenBergen, Norway

**Keywords:** hydrothermal systems, barite chimney, Epsilonproteobacteria, Loki's Castle Vent Field, chemolithoautotroph, low-temperature venting

## Abstract

In order to fully understand the cycling of elements in hydrothermal systems it is critical to understand intra-field variations in geochemical and microbiological processes in both focused, high-temperature and diffuse, low-temperature areas. To reveal important causes and effects of this variation, we performed an extensive chemical and microbiological characterization of a low-temperature venting area in the Loki's Castle Vent Field (LCVF). This area, located at the flank of the large sulfide mound, is characterized by numerous chimney-like barite (BaSO_4_) structures (≤ 1 m high) covered with white cotton-like microbial mats. Results from geochemical analyses, microscopy (FISH, SEM), 16S rRNA gene amplicon-sequencing and metatranscriptomics were compared to results from previous analyses of biofilms growing on black smoker chimneys at LCVF. Based on our results, we constructed a conceptual model involving the geochemistry and microbiology in the LCVF. The model suggests that CH_4_ and H_2_S are important electron donors for microorganisms in both high-temperature and low-temperature areas, whereas the utilization of H_2_ seems restricted to high-temperature areas. This further implies that sub-seafloor processes can affect energy-landscapes, elemental cycling, and the metabolic activity of primary producers on the seafloor. In the cotton-like microbial mats on top of the active barite chimneys, a unique network of single cells of Epsilonproteobacteria interconnected by threads of extracellular polymeric substances (EPS) was seen, differing significantly from the long filamentous *Sulfurovum* filaments observed in biofilms on the black smokers. This network also induced nucleation of barite crystals and is suggested to play an essential role in the formation of the microbial mats and the chimneys. Furthermore, it illustrates variations in how different genera of Epsilonproteobacteria colonize and position cells in different vent fluid mixing zones within a vent field. This may be related to niche-specific physical characteristics. Altogether, the model provides a reference for future studies and illustrates the importance of systematic comparative studies of spatially closely connected niches in order to fully understand the geomicrobiology of hydrothermal systems.

## Introduction

In hydrothermal vents, the microbial communities derive their energy from the chemical disequilibria that form when reduced hydrothermal fluids, rich in potential electron donors (e.g., H_2_, CH_4_, H_2_S, ${\text{NH}}_{4}^{+}$, and Fe^2+^), mix with seawater. These disequilibria support primary production by diverse chemolithoautotrophic microorganisms (Nakagawa and Takai, 2008; Kato et al., 2012; Sievert and Vetriani, 2012). Hydrothermal vent fields include multiple zones of focused, high-temperature venting and low-temperature, diffusing flows (Nakamura and Takai, 2014), where there are distinct geological settings that influence the fluid composition as well as the extent of venting on both spatial and temporal scales (Thornburg et al., 2010). The distribution of metabolically functional groups of microorganisms associated with high-temperature venting chimneys seems to be largely shaped by vent-specific energy landscapes formed by rapid mixing between hydrothermal fluids and seawater (McCollom and Shock, 1997; Amend et al., 2011; Kato et al., 2012; Dahle et al., 2015). Biotic and abiotic chemical processes are presumed to affect these energy landscapes only to a minor extent, given the typical high fluid flow through extremely sharp temperature and chemical gradients. Low-temperature, diffusing flows are however, the product of complex subseafloor processes, including seawater-hydrothermal fluid mixing, conductive cooling, redox reactions, and mineral precipitation (Nakamura and Takai, 2014). Hence, energy availabilities within low-temperature flow environments, as well as between high-temperature and low-temperature venting sites, can be expected to differ widely, even though the hydrothermal fluids originate from the same reservoir. Furthermore, the attenuated fluid flow regimes can also be hypothesized to affect the functions and adaptations in the residing microbial communities. Thus, a comprehensive understanding of the entire hydrothermal system is necessary in order to understand and assess the energy availabilities and microbial adaptations in low-temperature, diffusing flow sites compared to focused, high-temperature flow sites.

The Loki's Castle Vent Field (LCVF), located at the Arctic Mid-Ocean Ridge (AMOR) in the Norwegian-Greenland Sea, is a sediment-influenced, basalt-hosted hydrothermal field with emanating fluids with high concentrations of H_2_S, H_2_, CH_4_, and ${\text{NH}}_{4}^{+}$ (Pedersen et al., 2010). Consistently, Epsilonproteobacteria that oxidize H_2_ or H_2_S form dense biofilms on the black smokers in the LCVF (Dahle et al., 2013; Stokke et al., 2015). Furthermore, thermodynamic models of the LCVF suggest that this vent field represents an extremity in terms of its energetic potential for hosting anaerobic and aerobic methane oxidizers as well as aerobic ammonium oxidizers (Dahle et al., 2015). Congruently, in addition to biofilms dominated by Epsilonproteobacteria, biofilms with a dominance of aerobic methane oxidizers are identified on the black smoker walls (Dahle et al., 2015). However, in spite of ammonium oxidation being a potent potential energy source at LCVF, the microbial community composition of the biofilms did not confirm ${\text{NH}}_{4}^{+}$-based chemoautotrophy.

In a low-temperature flow area at the northeastern flank of the large sulfide mound at the LCVF, large cotton-like microbial mats cover unique, actively venting structures of nearly pure barite (BaSO_4_) (Pedersen et al., 2010). The venting fluids are diluted and chemically modified relative to the emission from the black smokers in the LCVF (Eickmann et al., 2014). A S-isotopic composition of the barite that is heavier than that of seawater suggests subsurface dissimilatory sulfate reduction, which may possibly be fueled by H_2_ or CH_4_ (Eickmann et al., 2014).

To achieve a better understanding of how these intra-field differences shape the structure, functions and adaptations of the microbial communities in the LCVF, we characterized microbial communities in microbial mats, barite chimney sections, and surrounding hydrothermal sediment in the diffuse, low-temperature venting area using microscopy, metatranscriptomics and 16S rRNA analyses. Through comparisons with observations from the high-temperature, focused black smokers (Dahle et al., 2013, 2015; Stokke et al., 2015), we developed a conceptual model of the geochemistry and microbiology of the entire hydrothermal system. Our model illustrates how energy landscapes, metabolic activity and adaptations in a hydrothermal system are affected by differences in fluid flows and chemical and microbiological alteration of the fluids.

## Materials and methods

**Figure 1:**
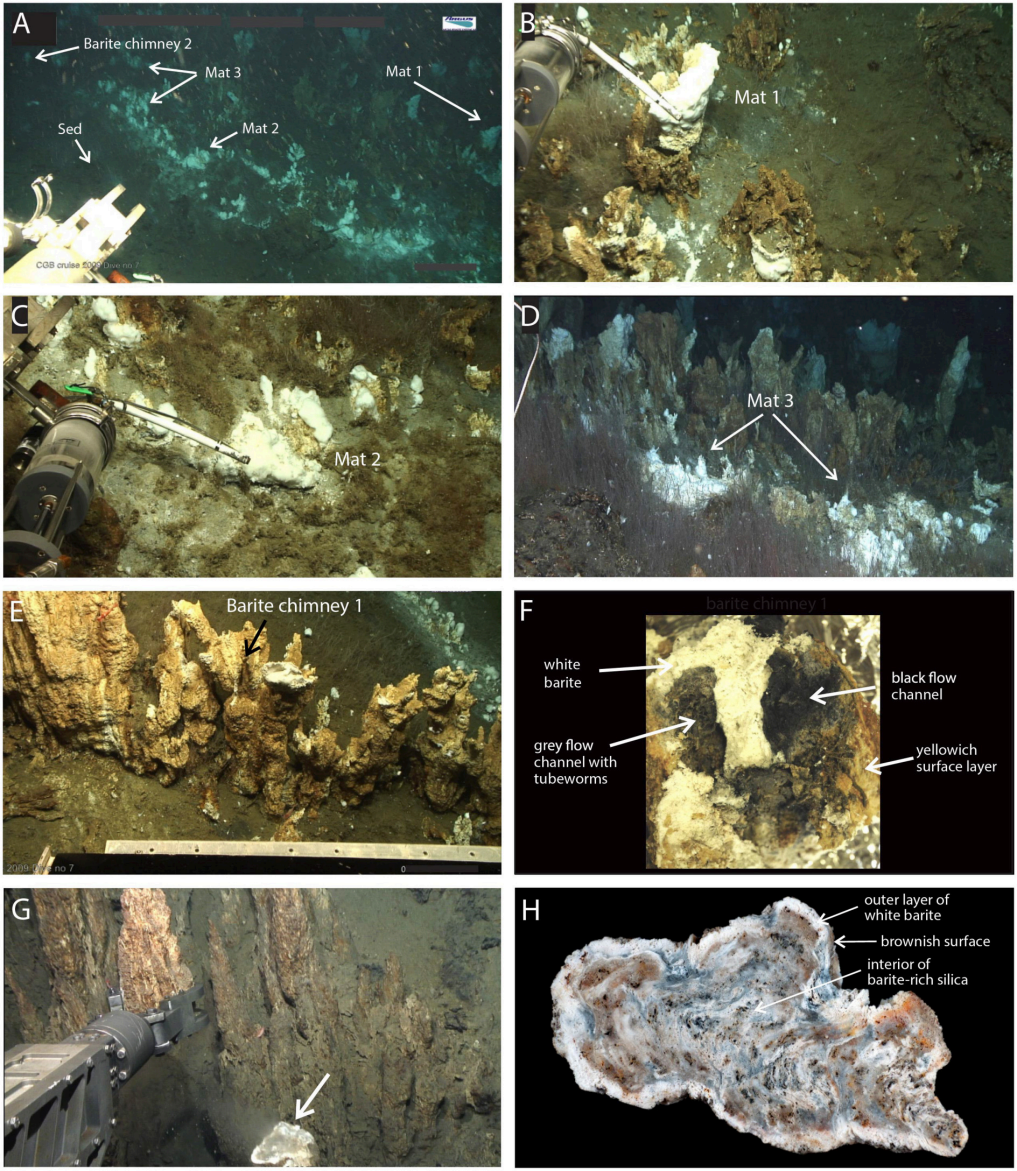
**Still images and sampling of the study sites**. Three microbial mats, Mat1-3, **(A–D)**, two active barite chimneys, BaCh1 **(E,F)** and BaCh2 **(A)** and an inactive barite silica chimney, SiCh **(G,H)** were sampled.

The LCVF is located on a volcanic ridge in the rift valley of the AMOR at the transition between the Mohns Ridge and the Knipovich Ridge at 73°30′N and 08°09′E and at a depth of 2400 m (Figure S1A) (Pedersen et al., 2010). The diffuse, low-temperature venting area on the northeastern flank of the hydrothermal mound (Figure S1B) is characterized by patchy dense colonization by siboglinid tubeworms (*Sclerolinum contortum*) (Kongsrud and Rapp, 2012) and small mound- to chimney-like structures (≤ 1 m tall) of barite (Figures 1A–E), which demonstrate active venting by being partially covered by thick (several cm), white microbial mats (Figures 1A–D; Figure S2A). The clear, shimmering fluids emanating from the barite structures and microbial mats have a temperature of 20°C (Pedersen et al., 2010). Their geochemical composition indicates subseafloor mixing of at least 10% high-temperature (320°C) hydrothermal fluids and cold (−0.7°C) percolating seawater, combined with subsequent modification due to microbial sulfate reduction (Eickmann et al., 2014). In addition to the active venting barite field, an extinct vent area with barite-rich silica chimneys (Figure 1G; Figure S2B) is present further to the southwest of the hydrothermal mound (Figure S1B).

### Sampling

A *Bathysaurus* ROV (Argus Remote Systems AS) equipped with video facilities was used to collect samples during research cruises to the LCVF in 2009 and 2010 with the research vessel G.O. Sars. From the barite field we collected three microbial mats using a 1 L hydraulic sampling cylinder (biosyringe), and two barite chimneys and one sediment sample using an aluminium scuffle box (Figure 1). Two of the mats (Mat2 and Mat3) were from small mound-like barite deposits and one (Mat1) was from a taller barite chimney. The samples were collected from the same area located at 73°33.99′N and 08°09.7′E, at a water depth of 2392 m, and about 0.5–1 m apart from each other (Figure 1A). A small barite chimney without a distinct flow channel (BaCh2) and hydrothermal sediment densely populated with *S. contortum* were also collected from this area. In addition, one branched barite chimney (BaCh1) containing two distinct central flow channels with gray to black colored walls (Figure 1F) was collected several meters (73°33.99′N and 08°09.72′E) away from the other samples (Figure 1B). For comparison, we also collected one barite-rich silica chimney (SiCh) from the extinct vent area (Figure 1G) at 73°33.99′N and 08°09.58′E at a water depth of 2367 m.

An overview of examined mat-samples and chimney sub-samples, respectively, are given in Table 1. From the BaCh1 chimney, the following samples were collected: gray (BaCh1GC) and black (BaCh1BC) material from the flow channels, white barite from the chimney wall interior (BaCh1W), and the light yellowish exterior (BaCh1O). From the BaCh2 chimney, the white interior (BaCh2W) and the yellowish exterior (BaCh2O) were sampled (Figure 1). Sub-samples of the sediment were taken from the rusty surface layer (SedRusty) and the black section below (SedBlack).

**Table 1:** **Description of samples, pyrosequencing depth of 16S rRNA PCR-amplicons and Shannon diversity indices**.

**Sample name**	**Description**	**Total clean reads^a^**	**Shannon^b^**
Mat1	Microbial mat	34562	2.82 (±0.10)
Mat2	Microbial mat	37692	3.88 (±0.08)
Mat3	Microbial mat	8685	3.91 (±0.07)
BaCh1W	Barite chimney1 interior	26058	4.45 (±0.05)
BaCh1GC	Gray barite chimney1 channel	48935	5.22 (±0.03)
BaCh1BC	Black barite chimney1 channel	36389	5.23 (±0.03)
BaCh1O	Barite chimney1 exterior	23499	3.91 (±0.07)
BaCh2W	Barite chimney2 interior	30798	4.30 (±0.05)
BaCh2O	Barite chimney2 exterior	47197	4.79 (±0.05)
SedRusty	Rusty surface sediment	77600	5.64 (±0.04)
SedBlack	Black sediment	5122	4.50 (±0.01)
SiChO	Barite-silica chimney exterior	11779	4.65 (±0.05)
SiChW	Barite-silica chimney white section	18540	5.03 (±0.04)
SiChC	Barite-silica chimney central section	23858	4.64 (±0.05)

a Total clean reads after pooling of samples according to Table S1.

b Calculated after subsampling of 5000 reads. Other subsampling depths are presented in Table S2.

Aliquots of the microbial mat samples were collected for microscopic examination. The cells in the remaining material were harvested by centrifugation for 6000 g for 5 min at 4°C. Mat samples and chimney and sediment sub-samples, respectively, were snap-frozen in liquid N_2_, and stored at −80°C.

### Scanning electron microscopy (SEM)

Sample material was fixed in 2.5% glutaraldehyde, collected on a 0.2 μm polycarbonate filter, dehydrated through a series of ethanol washes (10 min at 50%, 75%, 3 × 100%), air-dried, mounted on an aluminium specimen stub and coated with iridium in a Gatan 682 coater. The sample was studied by a Zeiss Supra 55VP field emission scanning electron microscope (FE-SEM; Carl Zeiss, Stockholm, Sweden), equipped with a Thermo Noran System SIX energy dispersive spectrometer (EDS) system (Carl Zeiss AS, Oslo, Norway).

### Fluorescence *In situ* hybridization (FISH) and DNA staining

FISH was performed on filters with fluorescently-labeled oligonucleotides (Glöckner et al., 1996). EPSY549 (5′-CAGTGATTCCGAGTAACG-3′) was labeled with Atto RHO 101 and used to target Epsilonproteobacteria (Lin et al., 2006). EPSY549Mod (5′-CAGTGATTCCGAATAACG-3′) was modified to target the identified *Sulfurimonas* phylotype and labeled with Atto RHO 101. NONEUB338 probes were labeled with Atto RHO 101 and used as control for non-specific staining (Christensen et al., 1999). Hybridizations were performed at 30% formamide for EPSY549 and at 20% formamide for EPSY549Mod. Fixed cells of *Escherichia coli* were used as a negative control. After the *in situ* hybridization, washing and drying, the cells were stained with the fluorescent DNA-binding dye 4′,6-diamidino-2-phenylindole (DAPI) (Morikawa and Yanagida, 1981). Stained slides were immersed in Immersol 518F (Carl Zeiss AG, Oberkochen, Germany) and evaluated in Zeiss Axio Imager Z1 microscope (Carl Zeiss Microscopy GmbH, Göttingen, Germany), equipped with filter 49 (DAPI), and 64 HE mPlum (Atto RHO 101).

### DNA extraction, PCR, and pyrosequencing

DNA was extracted from the microbial mats using the MasterPure™ Complete DNA and RNA Purification Kit (Epicentre Biotechnologies, Madison, WI, USA). The FastDNA spin kit for soil and the FastPrep® -24 Instrument (MP Biomedicals, Santa Ana, CA, USA) was used for DNA extractions of chimney and sediment sections. The amounts of nucleic acids were determined by A_260_ measurements using a Cary 300 Bio UV-Vis Spectrophotometer (Varian Inc., Palo Alto, CA, USA).

The 16S rRNA gene (the V5–V8 region) was amplified with the primers Un787f (5′-ATTAGATACCCNGGTAG) (Roesch et al., 2007) and Un1392r (5′-ACGGGCGGTGWGTRC) modified from Lane et al. (1985). The amplicons were generated in a two-step PCR using 1 × HotStar Taq® Master Mix Kit (Qiagen, Hilden, Germany), as previously described (Roalkvam et al., 2011; Jørgensen et al., 2012). Triplicate PCRs were conducted for each sample in the first step, and PCR products from each sample were pooled, purified using the MinElute PCR purification kit (Qiagen, Hilden, Germany) and then barcoded in the second step. The final PCR products were purified using Agencourt AMPure XP system (Beckman Coulter, Brea, CA, USA), and normalized amplicons from each sample were then pooled in a 1:1 ratio, comprising 20 ng each in the final suspension. Pyrosequencing of in total 33 subsamples (Table S1) was performed at the Norwegian Sequencing Center (Oslo, Norway) using the 454 FLX sequencer (Roche, Basel, Switzerland) with Titanium chemistry.

### cDNA synthesis

The cDNA synthesis was performed using SuperScript Double-Stranded cDNA synthesis kit (Invitrogen, Carlsbad, MA, USA) with added random hexamer primers (Thermo Fischer Scientific, Waltham, MA, USA). The RNA used was extracted simultaneous with DNA of Mat1 using the MasterPure™ Complete DNA and RNA Purification Kit (Epicentre, Madison, WI, USA). The MinElute PCR purification kit (Qiagen, Hilden, Germany) was used for sample clean-up and concentration. The cDNA protocol was implemented using triplicate samples that were later pooled and concentrated using Eppendorf concentrator 5301 (Eppendorf, Hamburg, Germany). In total, 673 ng of double stranded cDNA, as measured by SYBR-Green quantification (Roalkvam et al., 2011) was subjected to pyrosequencing at the Norwegian Sequencing Center (Oslo, Norway) using the 454 FLX sequencer (Roche, Basel, Switzerland) with Titanium chemistry.

### cDNA filtering and analyses

cDNA reads were filtered in MOTHUR (Schloss et al., 2009) using the trim.seqs command for removal of reads with at least one ambiguous nucleotide (maxambig = 0) or an average quality score at or below 25 (qaverage = 25). With these settings 247,763 out of the total 270,356 transcripts (91.6%) were retained for further downstream analyses. Filtered cDNA reads were compared to SSU and LSU rRNA gene sequences retrieved from the National Center for Biotechnological Information (NCBI) (http://www.ncbi.nlm.nih.gov/), using BLASTN (Altschul et al., 1997). In total, 237,719 transcripts were identified as rRNA from hits with a bitscore of ≥50. Among the remaining 10,044 reads, 5605 were identified as transcripts of genes with known function in MG-RAST (Meyer et al., 2008) (http://metagenomics.anl.gov/), and denoted as putative mRNA reads.

In addition, mRNA transcripts were analyzed in MG-RAST (Meyer et al., 2008) for taxonomic assignments, using the lowest common ancestor (LCA) algorithm and for functional annotation using default cutoff values (minimum *e*-value cutoff: 1e–5; minimum identity cutoff: 60%; minimum alignment length cutoff: 15).

### Taxonomic classification, hierarchical clustering, and diversity

Taxonomic assignments on filtered amplicon reads and rRNA reads were done in CREST (Lanzén et al., 2012), using the LCA algorithm on output from BLASTN (Altschul et al., 1997) searches against the SilvaMod SSURef database (Lanzén et al., 2012). The analyses were performed using default values except that no identity filter was chosen for the rRNA reads (option -f). In order to obtain reliable taxonomic assignments the default bitscore treshold of 155 was applied in both cases.

Filtered amplicons were clustered into operational taxonomic units (OTUs) using scripts distributed with AMPLICONNOISE (Quince et al., 2011). OTU clustering was performed with the maximum linkage clustering algorithm and a 3% difference cutoff. The resulting OTU table was analysed further with the VEGAN package of R (Oksanen et al., 2011) where Bray-Curtis distances (command “vegdist”) were calculated from relative OTU abundances (command “decostand”). Hierarchal clusters were constructed from the Bray-Curtis matrix using average linking (command “hclust”). Highly similar samples, as revealed by cluster analyses (see Results section), were treated as biological replicates and pooled in a modified OTU table prior to rarefaction analyses (command “rarefy”). Sample diversities were compared using in -house bash, python, and R scripts (available upon request) using a procedure were a constant number of reads were sampled randomly from each concatenated set of filtered reads followed by OTU clustering as described above and calculation of Shannon diversity indices in R. Standard deviations were based on results from 100 independently subsampled datasets.

### Deposition of sequence data

Thirty four raw sequence files (Table S4) have been submitted to the Sequence Read Archive (SRA310650) under the Bioproject PRJNA286711 and Biosample SAMN03765700.

## Results

### Microscopy and visible inspection

**Figure 2:**
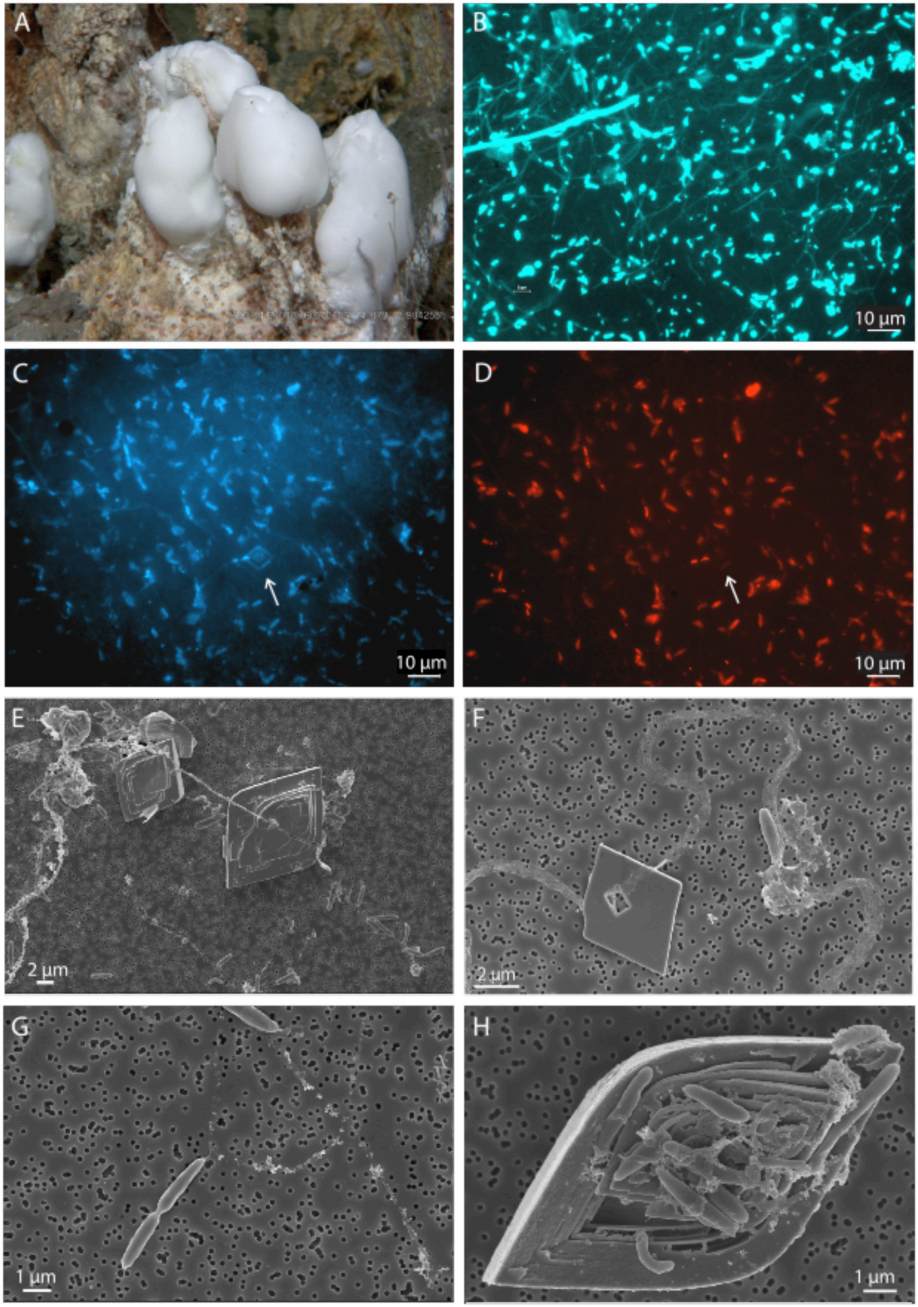
**Networks of cells and mineral interactions in a microbial mat on an active barite chimney**. Still photo of a microbial mat as it appeared on an active barite chimney **(A)**. DAPI photomicrograph showing networks of single rod-shaped cells connected with thin threads of extracellular substances **(B)**. Cells in the network were dominated by Epsilonproteobacteria of the genus *Sulfurimonas*, as visualized by DAPI and FISH using the EPSY549Mod probe. Arrows point to an attached barite crystal. **(C,D)** SEM images showing higher magnification of barite crystals developed on the threads **(E,F)**, a dividing cell attached to a thread **(G)**, and a defect, cavernous barite crystal due to the attachment and growth of a cell-aggregate **(H)**.

DAPI and FISH analyses showed that 2–3 μm long rod-shaped Epsilonproteobacteria of the genus *Sulfurimonas* dominated the microbial mats on top of the barite chimneys (Figures 2A–D). Most cells were polarly attached to and interconnected by thin (~200 nm) threads of extracellular polymeric substances (EPS) that were up to at least 100 μm long (Figures 2B–F). Occasionally, dividing cells were observed (Figure 2G). In addition to the attached cells, numerous small (up to 10 μm) barite crystals had nucleated and developed on the threads (Figures 2E,F). The attached crystals displayed characteristic cavities around the threads. Barite crystals with clusters of attached cells as well as numerous cavities and irregular growth defects were also abundant (Figure 2H).

### Structure of microbial communities

In total, 378,175 clean amplicon reads were obtained after filtering, ranging from 2078 to 34,914 per subsample (Table S1). Hierarchical clustering revealed distinct clusters of samples from the active barite chimneys, associated microbial mats, surrounding sediments as well as the inactive barite-rich silica chimney (Figure S3). Subsamples within each cluster were pooled prior to further analyses (Table S2). The microbial mats (Mat1-3) had the lowest diversity, while the highest diversity was observed in the rusty sediment (SedRusty) and the gray barite chimney channel (BaCh1GC) (Table 1). Rarefaction analyses gave congruent results, but revealed that only in the microbial mats and the yellow barite chimney exterior, the sampling was near complete (Figure S4).

**Figure 3:**
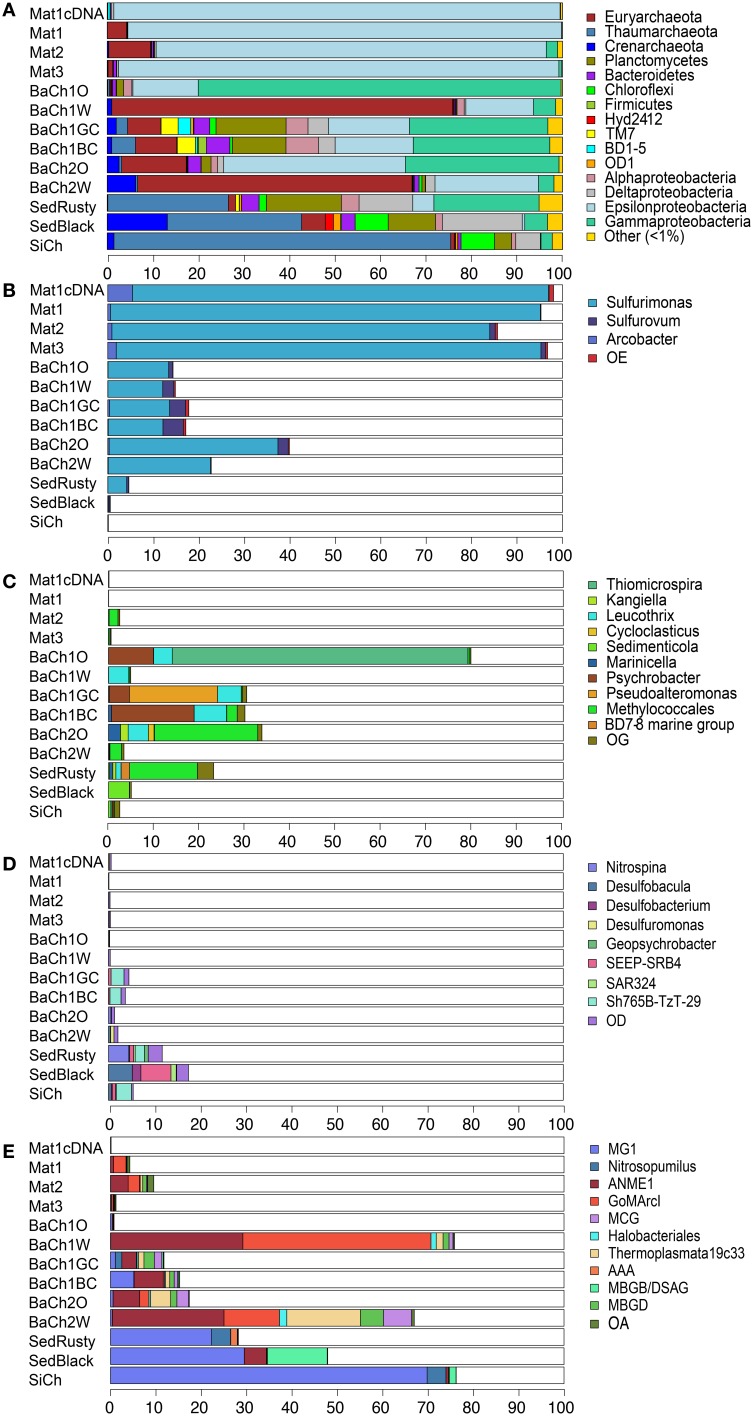
**Relative abundance of dominant microbial taxa in microbial mats (Mat1-3), subsamples of active barite chimneys (BaCh), sediments horizons (SedRusty, SedBlack) and in an inactive barite-silica chimney (SiCh) determined by pyrosequencing of 16S rRNA PCR amplicons or cDNA (Mat1cDNA)**. Phyla that represent >1% of the reads in at least one of the sites **(A)**. Epsilonproteobacterial genera **(B)**, Gammaprotebacterial genera **(C)**, Deltaproteobacterial genera **(D)**, and Archaea lineages **(E)**. Proteobacteria is in **(A)** presented on class level. OE, OG, OD, and OA refer to other Epsilonproteobacteria, Gammaproteobacteria, Deltaproteobacteria, or Archaea, respectively. A complete overview of the abundances of detected taxa is presented in Figure S5.

In the microbial mats (Mat1-3) and the active barite chimneys (BaCh1, BaCh2), the most abundant taxa were the proteobacterial classes Epsilonproteobacteria and Gammaproteobacteria, and the archaeal phylum Euryarchaeota (Figure 3A). In contrast, Thaumarchaeota predominated (80%) in the inactive silica chimney (SiCh), and was also abundant in the sediment sections (SedRusty, SedBlack). Furthermore, Crenarchaota, Planctomycetes, Bacteroidetes, Chloroflexi, Firmicutes and the Bacterial Candidate divisions TM7, BD1-5, OD1, and Hyd24-12 were also observed in abundances of >1% (Figure 3A). These taxonomic groups were most frequent in the gray and black flow channels (BaCh1GC, BaCh1BC) of the active barite chimneys and in the sediment.

The microbial mats were highly dominated by Epsilonproteobacteria (85–98%) where *Sulfurimonas* was by far the most abundant genus (Figure 3B). *Sulfurimonas* also occurred in high abundance in the sections of the barite chimneys BaCh1 (12.1–14.4%) and BaCh2 (22.5–37.0%), whereas in the sediment *Sulfurimonas* comprised a minor fraction of the community. The genus *Arcobacter* was also present in the microbial mats and *Sulfurovum* was identified in the barite chimney channels and the black sediment. Epsilonproteobacteria are widespread and account for a significant fraction of deep-sea vent chemoautotrophs (Campbell et al., 2006). Isolates of Epsilonproteobacteria are described as carrying out oxidation of H_2_ and sulfur compounds while reducing of oxygen, nitrate or sulfur species (Makita et al., 2012; Labrenz et al., 2013; Mino et al., 2014). Notably, Epsilonproteobacteria were absent in the inactive silica chimney (SiCh).

In the white interiors of the barite chimneys (BaCh1W, BaCh2W), Methanomicrobia were highly abundant comprising 50.3 and 37.1%, of the total reads, respectively. The Methanomicrobia were of uncultured, anaerobic, methanotrophic archaea (ANME-1) and GOM Arc-1, except in the rusty sediment where they were of the AAA-subcluster (Figure 3E). ANME-1 was also present in the black sediment (SedBlack), but absent in the inactive silica chimney (SiCh).

In the active barite chimneys (BaCh1, BaCh2), a high proportion of Gammaproteobacteria was observed (Figure 3C). Most notable was the high proportion of *Thiomicrospira* in the exterior section of chimney 1 (BaCh1O). *Thiomicrospira* spp. isolated from hydrothermal vents utilizes sulfur compounds (Ruby et al., 1981; Takai et al., 2004) and H_2_ (Hansen and Perner, 2015). In the exterior section of chimney 2 (BaCh2O), Methylococcales was abundant (Figure 3C), and a similar proportion was observed in the rusty sediment (SedRusty). Methylococcales was also present in Mat2. Methylococcales have previously been found to predominate in a biofilm on a black smoker chimney at LCVF (Dahle et al., 2015). In the barite chimney flow channels (BaCh1GC, BaCh1BC) a high proportion of genera including organotrophs, presumably being involved in degradation of organic debris in deep-sea environments (Orcutt et al., 2011) were observed (Figure 3C). They were also detected in other sections of the active barite chimneys and in the sediment. The genus *Sedimenticola* was uniquely detected in the sediment, and included the thiotrophic endosymbiont of *S. contortum* (Lösekann et al., 2008).

The sediment and the inactive silica chimney (SiCh) had a high abundance of Thaumarchaeota, of which MGI was dominating (Figure 3E). In addition, a minor fraction of *Nitrosopumilus*, which includes ammonia-oxidizers (Stahl and de la Torre, 2012), was present in the inactive silica chimney and the rusty surface sediment (Figure 3E). Furthermore, the highest share of Deltaproteobacteria was observed in the sediments (Figure 3A), comprising 11.6 and 17.5% in the rusty and black horizons, respectively. Uncultivated clades and different genera of Desulfobacterales occurred in different abundances in the sediment sections (Figure 3D). The nitrite-oxidizing genus *Nitrospina* (Spieck and Lipski, 2011) and the uncultivated Sh765B-TzT-29 clade were dominant in the rusty sediment. In contrast, the genera *Desulfobacula, Desulfobacterium*, SEEP-SRB4 and the SAR324 clade (Marine group B) dominated the black sediment (Figure 3D). These taxa are capable of utilizing complex organic substrates, including different alkanes (Ahn et al., 2009; Kleindienst et al., 2014; Rabus, 2014; Sheik et al., 2014). The Sh765B-TzT-29 clade was also present in the barite channels (BaCh1GC and BaCh1BC) and in the silica chimney. Furthermore, both the sediment and the chimney channels samples included a high abundance of Planctomycetes (Figure 3A), comprising between 10.4 and 16.5% of total reads. The Planctomycetes reads were mainly from the family Planctomycetaceae (Figure S5). Unique for the rusty sediment was the presence of reads (1.7%) classified as *Candidatus* Scalindua within the Brocardiaceae that includes anammox bacteria (Schmid et al., 2003).

### Transcription profile in the microbial mat

Metatranscriptomic sequencing of Mat1 yielded 111,322 16S rRNA reads whereof 91.5% were taxonomically assigned to *Sulfurimonas* (Figure 3B; Table S3). From the protein-coding RNA, 4670 of 5545 reads were classified as Epsilonproteobacteria (Table S3). The protein-coding reads suggested a sulfur-based metabolism due to the expression of a sulfide-quinone reductase (SQR) catalyzing the oxidation of sulfide to polysulfide chains or elemental sulfur coupled to quinone (Arieli et al., 1994) and the subunits of the sulfur oxidation (SOX) system (Table 2) responsible for the complete oxidation of thiosulfate to sulfate (Friedrich et al., 2000; Ghosh and Dam, 2009). The SOX system can also catalyze the oxidation of H_2_S, elemental sulfur and sulfite (Rother et al., 2001). Transcripts encoding a periplasmic hydrogenase (HydA, HydB, HydC) or a cytoplasmic hydrogenase (Sievert et al., 2008) were not recovered. A cbb3-type cytochrome *c* oxidase was expressed, which in Epsilonproteobacteria may function in aerobic respiration under microaerophilic conditions (Sievert et al., 2008) or as an electron acceptor in oxygen scavenging, preventing oxidative stress (Grote et al., 2012). Transcripts encoding enzymes of the denitrification pathway were identified suggesting utilization of nitrate as a terminal electron-acceptor (Table 2).

**Table 2:** **Transcribed functions (MG-RAST) in a microbial mat (Mat1) located on a barite chimney and in a biofilm on a black smoker wall at the LCVF**.

**Function**	**Microbial mat (Barite chimney)**		**Biofilm (Black smoker)^a^**	
	**Number of reads**	**Relative abundance (%)**	**Number of reads**	**Relative abundance (%)**
RNA polymerase subunit B	46	0.83	25	0.42
SoxX, SoxA	4	0.07	7	0.12
SoxY, SoxZ	9	0.16	64	1.09
SoxB	1	0.02	2	0.03
SoxCD	7	0.13	7	0.12
Sulfide quinone reductase (SQR)	17	0.31	46	0.78
Cytochrome b	1	0.02	13	0.22
Hydrogenase^b^	0	0.00	65	1.11
Formate dehydrogenase	5	0.09	0	0.00
Cytochrome c oxidase (cbb3-type)	26	0.47	112	1.91
Cytochrome c oxidase (other than cbb3-type)	0	0.00	8	0.14
Nitrate reductase (nap)	31	0.56	92	1.57
Nitric oxide reductase (nor)	4	0.07	34	0.58
Nitrous oxide reductase (nos)	4	0.07	4	0.07
Cytochrome nitrite reductase (cdnir)	1	0.02	13	0.22
Ferredoxin nitrite reductase	0	0.00	2	0.03
ATP citrate lyase	27	0.49	46	0.78
Pyruvate:ferredoxin oxidoreductase	83	1.50	117	2.00
2-oxoglutarate:acceptor oxidoreductase	65	1.17	84	1.43
Fumarate reductase/succinate dehydrogenase	16	0.29	5	0.09
Sensor/signal histidine kinase	57	1.03	17	0.29
Flagellar proteins	94	1.70	18	0.31
Methyl-accepting chemotaxis proteins	97	1.75	1	0.02

a Data from (Dahle et al., 2013) (5860 protein-encoding reads, sample 09ROV3BS).

b Group 1 membrane-bound hydrogen-uptake NiFe hydrogenase (Stokke et al., 2015).

Carbon assimilation via the reductive citric acid cycle and further via gluconeogenesis (Sievert et al., 2008), was evident based on the presence of transcripts encoding enzymes allowing the citric acid cycle to operate in reverse and catalyzing the bypassing reactions of gluconeogenesis (Table 2). Transcripts of ribulose 1,5-bisphosphate carboxylase (RubisCO) were not detected. Transcripts encoding the major components of the flagellar apparatus, the two components chemostaxis system (Che) signal transduction system (Szurmant and Ordal, 2004) and proteins with PAS (Per-ARNT-Sim) domains were abundant (Table 2). PAS domains are important signaling modules that monitor changes in light, redox potential, oxygen and overall energy level of the cell and are combined with a variety of regulatory modules in multi domain proteins allowing a spectrum of cell responses to changes in the environmental conditions (Taylor and Zhulin, 1999). Among the transcripts encoding proteins with PAS domains, the Aer-like redox taxis sensors, CetB, and the associated transducer protein (CetA) which in *Campylobacter jejuni* are involved in energy taxis (Schweinitzer and Josenhans, 2010), were identified. Cyclic diguanylate, c-di-GMP, is considered as one of the most common and important bacterial second messengers and transcripts encoding cyclic diguanylyate synthase and/or phosphodiesterase (11 transcripts) responsible for synthesis and hydrolysis of this compound (Römling et al., 2013) were identified. The intracellular levels of c-di-GMP are modified and monitored in microorganisms and result in regulation of processes such as biofilm formation, motility and virulence as well as a number of other processes (Römling et al., 2013).

## Discussion

**Figure 4:**
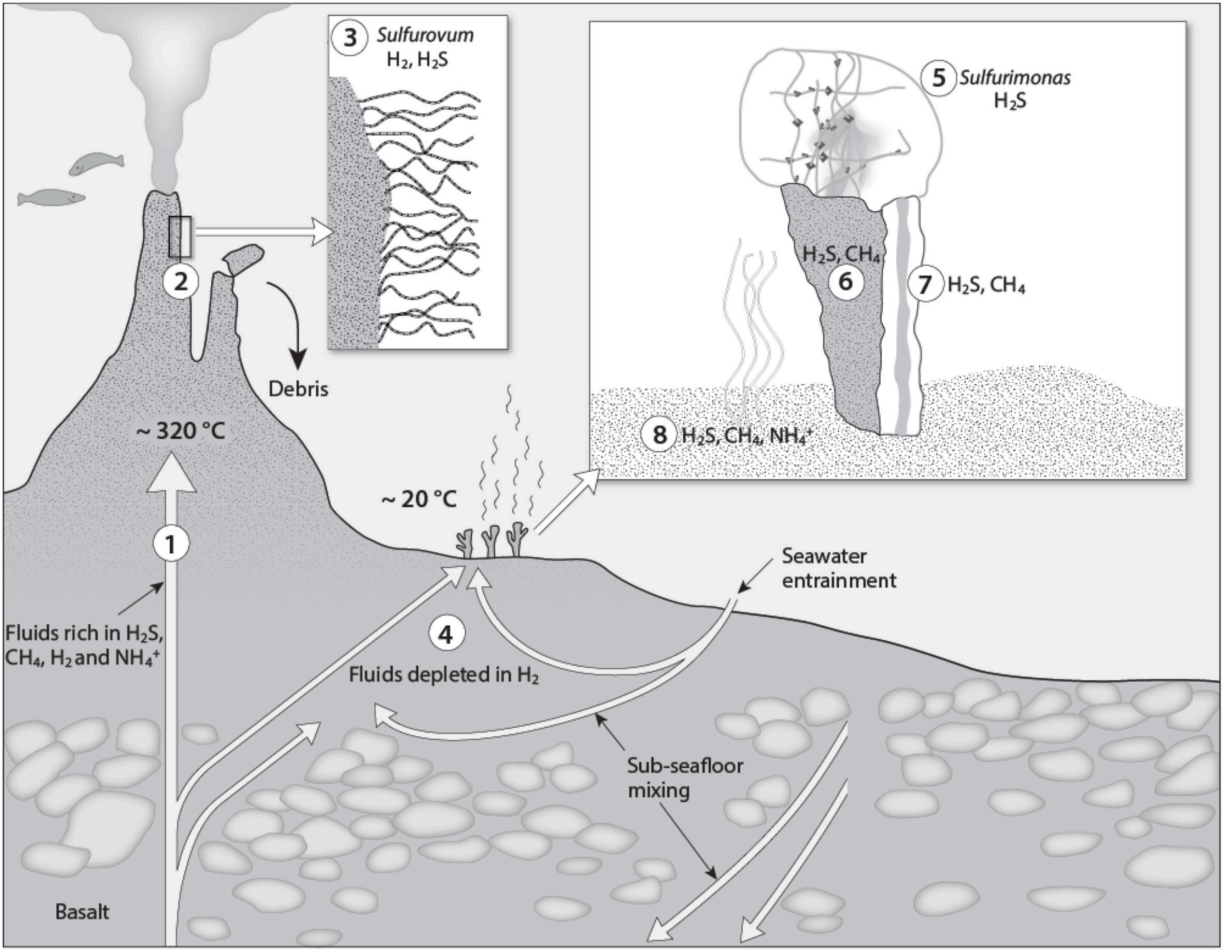
**A conceptual biogeochemical model of the Loki's Castle Vent Field**. (1) The high-temperature vent fluid is characterized by high CH_4_, H_2_, and ${\text{NH}}_{4}^{+}$ concentrations in addition to H_2_S (Pedersen et al., 2010). (2) The H_2_S, H_2_and CH_4_ support growth of Epsilonproteobacteria of the genera *Sulfurimonas* and *Sulfurovum* and gammaproteobacterial Methylococcales, respectively, in biofilms covering the black smoker chimneys (Dahle et al., 2013, 2015). (3) *Sulfurovum* forms large filamentous structures with sheaths of a heat resistant biopolymer (Stokke et al., 2015). (4) Subsurface mixing with percolating seawater and associated geochemical and microbial processes lead to sulfate reduction (Eickmann et al., 2014) and depletion of H_2_ in the low-temperature fluids discharged in the barite field. H_2_S in the diffuse venting fluids in the barite field supports microorganisms in the microbial mat on top of the barite chimneys (5), in the chimney exterior (6), interior (7), and in the hydrothermal sediment (8). CH_4_ supports microorganisms in the sediment and in the chimney interior and exterior, whereas ${\text{NH}}_{4}^{+}$ may be utilized in the surface sediment densely colonized by the tubeworm *Sclerolinum contortum* (Kongsrud and Rapp, 2012). The moderate fluid flow through the barite chimneys support biofilms of *Sulfurimonas* (5) forming delicate networks of single cells interconnected with EPS.

This study describes the microbial communities associated with a low-temperature, diffuse flow area of the LCVF and shows how physical and chemical differences between this site and the focused, high-temperature focused flow site within the same hydrothermal system correspond to differences in composition, spatial organization of cells in biofilms/mats and gene transcription profiles in the microbial communities they host. A conceptual model of the microbes associated with diffuse and focused venting is presented in Figure 4. The high-temperature fluids at LCVF are characterized by H_2_S concentrations in the range of 2.6–4.7 mmol kg^−1^, as well as high CH_4_ (12.5–15.5 mmol kg^−1^), H_2_ (4.7–5.5 mmol kg^−1^), ${\text{NH}}_{4}^{+}$, (4.7–6.1 mmol kg^−1^) and CO_2_ (22.3–26.0 mmol kg^−1^) concentrations (Pedersen et al., 2010). The low-temperature fluids venting from barite chimneys at the flank of the hydrothermal mound are, however, diluted and chemically modified relative to the focused emission from the black smokers. Measurements of the ammonium concentrations (600 μM) in the fluid emitted through the barite chimneys indicate that these fluids were comparable to the focused flow fluids diluted by seawater in a 1:10 relationship (Eickmann et al., 2014). Microbial community models from modeled energy availabilities suggest that around this dilution factor (corresponding to a temperature of around 20°C), there is a transition from growth conditions favorable for anaerobic methane oxidizers to those favorable for aerobic sulfide and methane oxidizers (Dahle et al., 2015). In agreement with this model, sulfide-oxidizing Epsilonproteobacteria (Table 2) of the genus *Sulfurimonas* were identified as the major chemolithoautotroph in the microbial mats on the active barite chimneys (Figures 2, 3). A high proportion of *Sulfurimonas* was also detected in the barite chimneys. In addition, sulfide- or methane-oxidizing Gammaproteobacteria, *Thiomicrospira* or Methylococcales, respectively, were abundant on the chimney exteriors. However, in the chimney interiors, ANME-1, were predominant. These groups were almost or completely absent in the inactive silica chimneys. Thus, as in the focused high-temperature venting site, CH_4_ and H_2_S are important electron donors influencing the energy landscape and energy availability seems to be a good indicator for the abundance of microbial functional groups not only in focused flow systems, as previously demonstrated (Dahle et al., 2015), but also in diffuse flow environments. The model suggests, however, that H_2_ present at high concentrations in the high-temperature “source” fluid is consumed subsurface, after mixing with percolating seawater, resulting in emissions of low-temperature H_2_ depleted fluids in the barite field. This inference is demonstrated in the isotopic signatures of the barite chimneys, suggesting subsurface microbial sulfate reduction (Eickmann et al., 2014). Moreover, CH_4_ but no H_2_ was detected in fluids emitted through a barite chimney and in a 2-m long sediment core from the barite field (T. Baumberger pers. comm.). Finally, hydrogenase transcripts were not detected in the *Sulfurimonas* dominated mat (Table 2) supporting the conclusion that H_2_ is not available as a substrate in the fluids venting through the barite chimneys. This contrasts with the result that in *Sulfurovum* mats growing on black smokers at LCVF, hydrogenases were highly transcribed (Dahle et al., 2013; Stokke et al., 2015). Whether the *Sulfurimonas* cells in the barite field lack hydrogenases in their genome or induce hydrogenase transcription only when H_2_ is available, as observed for *Sulfurovum* NBC37-1 (Yamamoto et al., 2010), remains to be investigated. Furthermore, it cannot be ruled out that the absence of hydrogenase transcripts is due to sample biases and the low number of protein-coding reads (5545) obtained from the mat. Yet, the *Sulfurimonas* growing in the barite field and the *Sulfurovum* associated with focused flow fluids, seemed to demonstrate a highly similar genetic basis for energy acquisition from oxidation of sulfur compounds (SOX and SQR genes) and denitrification (Nir, Nor, Nos). As previously noted for Epsilonproteobacteria, carbon-fixation by the *Sulfurimonas* population is mediated by use of the rTCA cycle and transcript of RubisCO known to operate in sulfur-oxidizing Gammaproteobacteria were not detected (Takai et al., 2005). Altogether, our data illustrate how microbially relevant energy landscapes at the seafloor may be influenced by subsurface processes.

ANME-1 is likely performing anaerobic methane-oxidation (AOM) (Knittel and Boetius, 2009; Holler et al., 2011), but ANME-1 is also found in net methane-producing sediments (Lloyd et al., 2011). Due to subsurface H_2_ depletion in the low-temperature fluids emitted through the barite chimneys, the role of ANME-1 in the barite chimneys seems to be related to AOM rather than methanogenesis. However, only a very low share (< 0.1%) of SEEP-SRB1 was identified in the barite chimney, indicating that the ANME-1 population was free-living as previously observed in Nyegga pockmarks (Roalkvam et al., 2011).

Neither the low-temperature venting barite chimneys (Figure 3) nor the high-temperature black smoker chimneys in LCVF seem to host ammonium oxidizers, despite the fact that ammonium oxidation has been shown, through modeling, to represent a relatively potent energy source (Dahle et al., 2015). This study detected members of *Candidatus* Scalinuda, which may grow by anaerobic ammonium oxidation, putative aerobically ammonium-oxidizing *Nitrosopumilus* as well as nitrite-oxidizing *Nitrospina* in the rusty surface sediment in the barite field. Furthermore, a high proportion of MGI was seen in the black sediment horizon. These results indicate that ammonium oxidation is in fact a biologically relevant energy source in LCVF and point to a possible hotspot for biological nitrogen cycling, in the sediments. However, it should be noted that a high fraction of MG1 was also seen in the distinct barite-silica chimneys. Furthermore, planctomycetes performing anammox are found to carry out additional metabolic properties (Strous et al., 2006; Oshiki et al., 2013). Thus, our results should be taken with some caution and clarification of the role of MG1 and *Candidatus* Scalindua in the nitrogen cycling at LCVF will ultimately require *in situ* rate measurements of nitrification and anammox.

Whereas energy availability may be key factors controlling the distribution of functional groups of organisms in the diffusely venting site in LCVF, our data also indicate that the distribution of specific ecotypes may be controlled by specific physical factors constraining the structuring of the microbial mats. The microbial mats situated on top of the barite chimneys consisted almost exclusively of members of *Sulfurimonas*, which where interconnected and polarly attached to ultrathin EPS threads (Figure 2). To our knowledge, this structure is unique and has so far not been reported for any other Epsilonproteobacteria. The EPS threads seemed to provide a backbone for the microbial mat structure allowing the cells to be positioned within the narrow redox gradient between the reduced venting fluid and the oxidized seawater. In order to make this possible, the specific density and structure of the interconnected cells must be rigid and heavy enough to withstand fluid flow forces and bottom currents, while at the same time allow the venting fluids to escape through the mat. In this respect, the barite crystals attached to the EPS threads may be regarded as an important component of the mats, as they increase the specific density of the structure, thereby allowing a less dense network of cells and EPS. Moreover, formations of such an advanced mat structure where the cells are positioned for an efficient acquisition of energy require that the bacteria detect changes in environmental conditions and respond by navigating toward niches that support optimal growth. Consistently, we observed expression of the flagellar components as well as methyl-accepting chemotaxis proteins (MCPs) and the core chemotaxis components CheA, CheW and CheY that transduce signals to the flagellar apparatus allowing motility responses to changes in chemical composition in the environment. Moreover, our data suggest that the cells also may have the capacity to respond to altered internal energetic conditions by involving sensors with PAS domains and enzymes that may influence the intracellular level of c-di-GMP. We did observe that the majority of the cells in the mat were sessile, attached to the EPS threads. However, we also observed that the cells linked to the EPS threads were dividing and we therefore speculate that motile cells are released and can navigate through the mat structure to more favorable metabolic niches. In summary, the *Sulfurimonas* mat structure appeared to represent a specific adaptation to life in this environment as it seems to position the cells optimally for an efficient acquisition of energy on top of the venting barite chimney. This point is made even more clearly when we compare the *Sulfurimonas* mats from this study with the mat comprising Epsilonproteobacteria of the *Sulfurovum* genus observed on a black smoker chimney wall of LCVF (Stokke et al., 2015). This *Sulfurovum*-dominated mat was made up of long filaments surrounded by thermotolerant sheaths, presumably composed of a polymer resembling chitin or cellulose. A similar structure was also observed for *Sulfurovum* epibionts growing on the anomuran crab, *Shinkaia crosnieri* (Watsuji et al., 2010, 2012), and is arguably more suited to attachment on solid surfaces adjacent to the vigorous focused hydrothermal fluid flows. The sessile lifestyle that the microorganisms experience in this type of mat situated in environments with a constant supply of hydrothermal fluids rich in electron donors, would be less dependent on environmental sensing, chemotaxis and motility, which is in line with our observations (Table 2). A third category of mat structures formed by Epsilonproteobacteria is represented by *Arcobacter*, forming sulfur-filaments (Sievert et al., 2007). Altogether, this illustrates that different ecotypes within the primary producing Epsilonproteobacteria not only may be differentiated by their energy metabolism, but that specific mat-formation adaptations depending on their physical surrounding can be equally or even more important. Such adaptations are not easily identified from genome information, emphasizing the importance of analysing the physical appearance of Epsilonproteobacteria in their natural environment in order to fully understand their diversity and distribution. Altogether, the data show how different genera of Epsilonproteobacteria can apply different strategies to colonize and position cells in mixing zones at focused and diffuse vents, which can be related to differences in flows rates and chemistry of the effluent fluids and the specific geographic location in the vent field.

## Author contributions

IS designed the experiments, conducted the research and wrote the paper. HD bioinformatic data analysis and writing. RS bioinformatic data analysis. IR lab work and writing. FD lab work and writing. HR environmental sampling and writing. RP environmental sampling and writing. IT lab work and writing of paper.

### Conflict of interest statement

The authors declare that the research was conducted in the absence of any commercial or financial relationships that could be construed as a potential conflict of interest.
