# Supplementary material for: Novel Barite Chimneys at the Loki's Castle Vent Field Shed Light on Key Factors Shaping Microbial Communities and Functions in Hydrothermal Systems
Source: Front Microbiol. 2016 Jan 7;6:1510. doi: 10.3389/fmicb.2015.01510 (PMC4703759; doi:10.3389/fmicb.2015.01510)
Supplement: Supplementary file 1 [file Table1.PDF]

Table S1. Statistics of 454 pyrosequencing data.

Sample name	Sub-sample	Raw reads	Reads after filtering	Share filtered out	Chimeric reads	Share chimeric reads	Clean reads	Unique clean	OTUs (97%)	Chao2 (OTUs)
Mat1	Mat1(A-MID16)	39045	4480	11.5%	3	0.0%	34562	116	94	145
Mat2	Mat2(A-MID7)	18698	1958	10.5%	8	0.0%	16732	223	179	355
Mat2	Mat2(A-MID21)	18698	2284	12.2%	8	0.0%	16406	187	144	230
Mat2	Mat2(B-MID17)	5858	5260	10.2%	706	13.4%	4554	344	276	571
Mat3	Mat1(B-MID1)	9995	8887	11.1%	202	2.3%	8685	203	156	341
BaCh1W	BaCh1W(A-MID2)	19072	3555	18.6%	96	0.6%	15421	426	348	636
BaCh1W	BaCh1W(B-MID23)	6164	5509	10.6%	1071	19.4%	4438	199	167	425
BaCh1W	BaCh1W(B-MID31)	3097	2706	12.6%	628	23.2%	2078	168	125	344
BaCh1W	BaCh1W(B-MID32)	5763	5058	12.2%	937	18.5%	4121	215	172	355
BaCh1GC	BaCh1GC(B-MID19)	29285	25652	12.4%	5450	21.2%	20202	1081	846	1518
BaCh1GC	BaCh1GC(B-MID20)	16253	14357	11.7%	3636	25.3%	10721	840	649	1397
BaCh1GC	BaCh1GC(B-MID21)	26369	23325	11.5%	5313	22.8%	18012	1038	823	1751
BaCh1BC	BaCh1BC(A-MID14)	14563	2763	19.0%	88	0.7%	11712	685	465	722
BaCh1BC	BaCh1BC(B-MID33)	31590	27964	11.5%	3287	11.8%	24677	1061	797	1278
BaCh1O	BaCh1O(B-MID22)	26661	23898	10.4%	399	1.7%	23499	338	257	435
BaCh2W	BaCh2W(B-MID2)	14289	11508	19.5%	51	0.4%	11457	358	295	532
BaCh2W	BaCh2W(B-MID3)	15685	13376	14.7%	49	0.4%	13327	363	302	558
BaCh2W	BaCh2W(B-MID4)	7164	6071	15.3%	57	0.9%	6014	287	229	418

BaCh2O	BaCh2O(B-MID5)	7448	6604	11.3%	22	0.3%	6582	361	286	497
BaCh2O	BaCh2O(B-MID6)	6361	5723	10.0%	22	0.4%	5701	314	240	380
BaCh2O	BaCh2O(B-MID7)	41911	36988	11.7%	2074	5.6%	34914	844	653	1170
SedRusty	SedRusty(A-MID11)	27633	5824	21.1%	488	2.2%	21321	1931	1330	2047
SedRusty	SedRusty(A-MID22)	29457	5937	20.2%	152	0.6%	23368	1490	1062	1581
SedRusty	SedRusty(B-MID35)	37427	33066	11.7%	155	0.5%	32911	1823	1247	1853
SedBlack	SedBlack(A-MID17)	6509	1380	21.2%	7	0.1%	5122	179	156	257
SiCh	SiChO(B-MID9)	6813	6055	11.1%	52	0.9%	6003	490	371	735
SiCh	SiChO(B-MID10)	6469	5831	9.9%	55	0.9%	5776	351	279	711
SiCh	SiChW(B-MID11)	8165	7243	11.3%	54	0.7%	7189	672	532	955
SiCh	SiChW(B-MID12)	5237	4684	10.6%	7	0.1%	4677	407	331	627
SiCh	SiChW(B-MID13)	7512	6697	10.8%	23	0.3%	6674	458	358	706
SiCh	SiChC(B-MID14)	15197	13769	9.4%	549	4.0%	13220	519	430	911
SiCh	SiChC(B-MID15)	6852	6186	9.7%	224	3.6%	5962	422	359	761
SiCh	SiChC(B-MID16)	5368	4793	10.7%	117	2.4%	4676	451	366	810
