# Supplementary material for: Novel Barite Chimneys at the Loki's Castle Vent Field Shed Light on Key Factors Shaping Microbial Communities and Functions in Hydrothermal Systems
Source: Front Microbiol. 2016 Jan 7;6:1510. doi: 10.3389/fmicb.2015.01510 (PMC4703759; doi:10.3389/fmicb.2015.01510)
Supplement: Supplementary file 2 [file Table2.PDF]

Table S2. Shannon indices at different sampling depths.

Sample name	Molecular Identifier (MID)	Total clean reads	Mean (Shannon) 500	SD_500	Mean (Shannon) 2000	SD_2000	Mean (Shannon) 5000	SD_5000	Mean (Shannon) 10000	SD_10000
Mat1	B-MID01	8685	2.415	0.138	3.490	0.096	3.914	0.070	4.225	0.044
Mat2	A-MID07, A-MID21, B-MID17	37692	2.522	0.121	3.366	0.105	3.876	0.075	4.253	0.063
Mat3	A-MID16	34562	1.824	0.179	2.393	0.170	2.816	0.101	2.935	0.080
BaCh1_W	A-MID2, B-MID23, B-MID31, B-MID32	26058	3.306	0.088	3.905	0.065	4.454	0.052	4.794	0.046
BaCh1_GC	B-MID19, B-MID20, B-MID21	48935	4.098	0.060	4.694	0.037	5.219	0.032	5.491	0.029
BaCh1_BC	A-MID14, B-MID33	36389	3.948	0.072	4.724	0.045	5.231	0.034	5.472	0.028
BaCh1_O	B-MID22	23499	2.425	0.125	3.498	0.110	3.912	0.066	4.251	0.049
BaCh2_W	B-MID2, B-MID3, B-MID4	30798	3.165	0.086	3.893	0.071	4.301	0.052	4.865	0.043
BaCh2_O	B-MID5, B-MID6, B-MID7	47197	3.507	0.086	4.001	0.071	4.789	0.049	5.087	0.037
Sed_Rusty	A-MID11, A-MID22, B-MID35	77600	4.115	0.065	5.078	0.038	5.638	0.036	5.931	0.027
Sed_Black	A-MID17	5122	3.477	0.085	4.153	0.044	4.501	0.010		
SiCh_O	B-MID9, B-MID10	11779	2.746	0.151	3.902	0.092	4.651	0.054	5.088	0.022
SiCh_W	B-MID11, B-MID12, B-MID13	18540	3.341	0.099	4.314	0.085	5.030	0.040	5.423	0.023
SiCh_C	B-MID14, B-MID15, B-MID16	23858	2.888	0.129	3.994	0.076	4.644	0.049	4.914	0.039
