# Supplementary material for: Novel Barite Chimneys at the Loki's Castle Vent Field Shed Light on Key Factors Shaping Microbial Communities and Functions in Hydrothermal Systems
Source: Front Microbiol. 2016 Jan 7;6:1510. doi: 10.3389/fmicb.2015.01510 (PMC4703759; doi:10.3389/fmicb.2015.01510)
Supplement: Supplementary file 3 [file Table3.PDF]

Table S3. **cDNA sequencing data of Mat1.**

Total reads	270,356
Filtered reads	22,593
Clean reads[1]	247,763
rRNA reads [2]	238,719
16S rRNA reads[3]	111,322
non-rRNA reads	10,044
Protein coding RNA (MG-RAST)	5,545
Protein coding RNA classified by LCA as *Epsilonproteobacteria* (MG-RAST)	4,670

1 Clean reads = Total number of reads – filtered reads.
Numbers in rows below this point are based on clean reads only.
2 Reads with bitscore > 50 in BLASTX searches against the 5S, 16S, 23S and 18S rRNA genes.
3 Reads with bitscore > 150 in BLASTN searches against SilvaMod104.
4 Number of reads assembled using gsAssembler, Newbler version 2.8.
Reads with bitscore > 50 to NCBIs RefSeq protein database.
Reads with protein annotation in MG-RAST using an e-value treshold of 10^{-5}.
