# Supplementary material for: Novel Barite Chimneys at the Loki's Castle Vent Field Shed Light on Key Factors Shaping Microbial Communities and Functions in Hydrothermal Systems
Source: Front Microbiol. 2016 Jan 7;6:1510. doi: 10.3389/fmicb.2015.01510 (PMC4703759; doi:10.3389/fmicb.2015.01510)
Supplement: Supplementary file 4 [file Table4.PDF]

Table S4. Overview of files deposited in SRA.

Sample name	Sub-sample	name of file in SRA	MD5 checksum
Mat1	Mat1(A-MID16)	Mat1_A_MID16.sff	95476a9175017c658f6af3ff41af4f
Mat2	Mat2(A-MID7)	Mat2_A_MID7.sff	f62b9c004e7f05eaa890e0d354382
Mat2	Mat2(A-MID21)	Mat2_A_MID21.sff	a521bc6f65a6a77480b879847ad82
Mat2	Mat2(B-MID17)	Mat2_B_MID17.sff	3918e5ff11fd84ef2d4bd1a8e870de
Mat3	Mat1(B-MID1)	Mat1_B_MID1.sff	df7c07e3f99a46de1f6a18adf89e89
BaCh1W	BaCh1W(A-MID2)	BaCh1W_A_MID2.sff	29425c5e38dbd04ffc10df1c344cb2
BaCh1W	BaCh1W(B-MID23)	BaCh1W_B_MID23.sff	bf2a3223d1f0fbb18530302cd87b9
BaCh1W	BaCh1W(B-MID31)	BaCh1W_B_MID31.sff	57da3e9077e0197fb06372873b76e
BaCh1W	BaCh1W(B-MID32)	BaCh1W_B_MID32.sff	69e2bb33bca0ceb141fcf45ec3eae1
BaCh1GC	BaCh1GC(B-MID19)	BaCh1GC_B_MID19.sff	7acf773718d6d3d3212fef41abc791
BaCh1GC	BaCh1GC(B-MID20)	BaCh1GC_B_MID20.sff	6f98e90b327516164ac63fc577fd6
BaCh1GC	BaCh1GC(B-MID21)	BaCh1GC_B_MID21.sff	54ec5e3a2be18129b23de225b1800
BaCh1BC	BaCh1BC(A-MID14)	BaCh1BC_A_MID14.sff	625562e44e2833f7c749b97c42a57
BaCh1BC	BaCh1BC(B-MID33)	BaCh1BC_B_MID33.sff	fdfc2960e815a6dfb671028b12b35
BaCh1O	BaCh1O(B-MID22)	BaCh1O_B_MID22.sff	5964c6a82f60f496598dc0cf38664
BaCh2W	BaCh2W(B-MID2)	BaCh2W_B_MID2.sff	c9e638fc6c0b42b8c59ffc043f3d2a
BaCh2W	BaCh2W(B-MID3)	BaCh2W_B_MID3.sff	b86139646442c058f0c5d65f86b33
BaCh2W	BaCh2W(B-MID4)	BaCh2W_B_MID4.sff	7bd49175a131f9b84233e792942dd
BaCh2O	BaCh2O(B-MID5)	BaCh2O_B_MID5.sff	3467cc595173827aca643ff62e590
BaCh2O	BaCh2O(B-MID6)	BaCh2O_B_MID6.sff	0166efa12602434aeaa2e79c1322fb
BaCh2O	BaCh2O(B-MID7)	BaCh2O_B_MID7.sff	dda529bf09e94be141b82d4d9d9ca
SedRusty	SedRusty(A-MID11)	SedRusty_A_MID11.sff	176c244afd280aa5f564c17b64179f
SedRusty	SedRusty(A-MID22)	SedRusty_A_MID22.sff	718be08accfa3fd96905e1e3e2e753
SedRusty	SedRusty(B-MID35)	SedRusty_B_MID35.sff	7f82675482d62c1154e4dae3e2f40
SedBlack	SedBlack(A-MID17)	SedBlack_A_MID17.sff	b6683523302135a4ebf6c71eb4490
SiCh	SiChO(B-MID9)	SiChO_B_MID9.sff	c12ddcaf3c6d92f8bee06d599f9938

SiCh	SiChO(B-MID10)	SiChO_B_MID10.sff	0cd14461a00a968bab4e1c9fb6075
SiCh	SiChW(B-MID11)	SiChO_B_MID11.sff	3424d0893ef299e1ba703cdcdc817
SiCh	SiChW(B-MID12)	SiChW_B_MID12.sff	442bdbdf316b7ab16d44d1a337242
SiCh	SiChW(B-MID13)	SiChW_B_MID13.sff	c8736f2451f592b93023c8f6b6de5
SiCh	SiChC(B-MID14)	SiChC_B_MID14.sff	4a8f0851960f53d044569cb2e1538
SiCh	SiChC(B-MID15)	SiChC_B_MID15.sff	e5ba8bb517ec20fa645822230ec48
SiCh	SiChC(B-MID16)	SiChC_B_MID16.sff	64801c2145d51a0bf04cbcfe7360e
Mat1cDNA	-	09ROV8_09_cDNA.sff	f029ea5d95354460fb7d90af93734
