# Supplementary material for: Novel Barite Chimneys at the Loki's Castle Vent Field Shed Light on Key Factors Shaping Microbial Communities and Functions in Hydrothermal Systems
Source: Front Microbiol. 2016 Jan 7;6:1510. doi: 10.3389/fmicb.2015.01510 (PMC4703759; doi:10.3389/fmicb.2015.01510)
Supplement: Supplementary file 5 [file DataSheet1.docx]

**Supporting information**

**FIGURE S1**. **Sampling location.** A simplified map of the Arctic Mid-Ocean Ridge showing the location of the Loki´s Castle Vent Field as indicated with a red dot **(A)**. Bathymetric map of the Loki´s Castle hydrothermal mound showing the location of the barite field and the extinct barite-silica chimneys relative to the black smoker chimneys **(B)**.

**FIGURE S2. Study sites.** Active barite chimneys partially covered by white microbial mats **(A)**. Extinct barite-rich silica chimneys **(B)**.

**FIGURE S3. Dendrogram of subsamples resulting from hierarchical average linkage clustering based on Bray-Curtis dissimilarities between OTU-abundances.**

**FIGURE S4. Rarefaction analyses of the number of OTUs found in each of the datasets (concatenated according to Table 1).**

**FIGURE S5. Relative taxon abundance at different ranks.**

**Table S1. Statistics of 454 pyrosequencing data.**

**Table S2. Shannon indices at different sampling depths.**

**Table S3. cDNA sequencing data of Mat1.**

**Table S4. Overview of files deposited in SRA.**
