# Supplementary figures and images for: Novel Barite Chimneys at the Loki's Castle Vent Field Shed Light on Key Factors Shaping Microbial Communities and Functions in Hydrothermal Systems
Source: Front Microbiol. 2016 Jan 7;6:1510. doi: 10.3389/fmicb.2015.01510 (PMC4703759; doi:10.3389/fmicb.2015.01510)

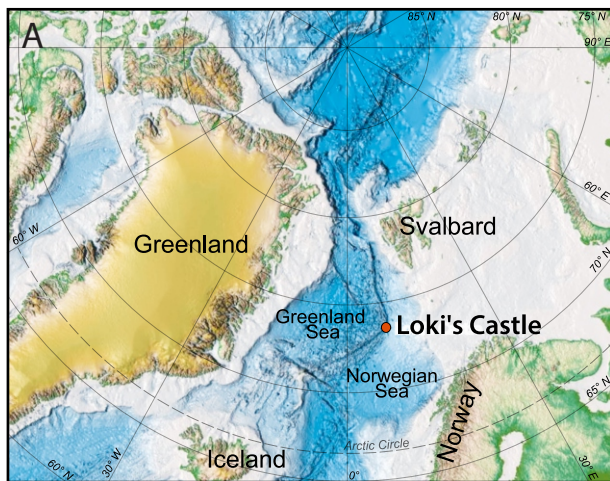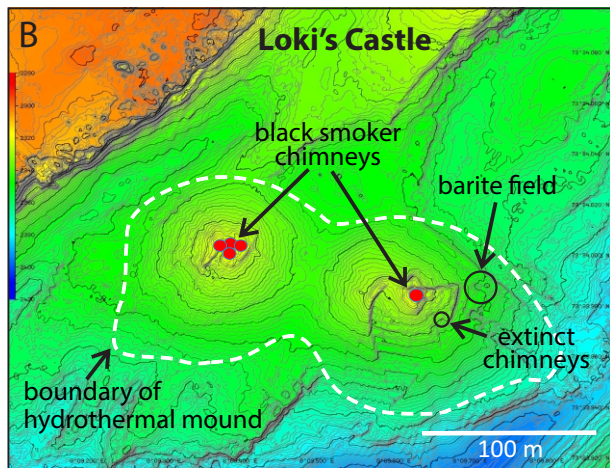

Supplement: Supplementary file 6 [file Image1.pdf]

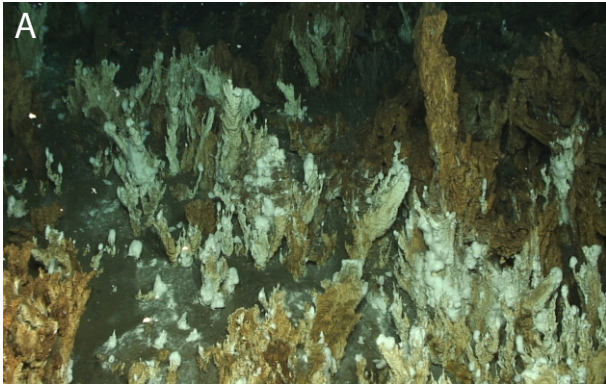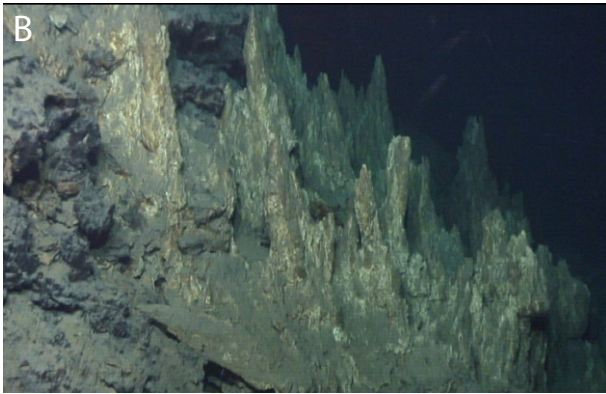

Supplement: Supplementary file 7 [file Image2.pdf]

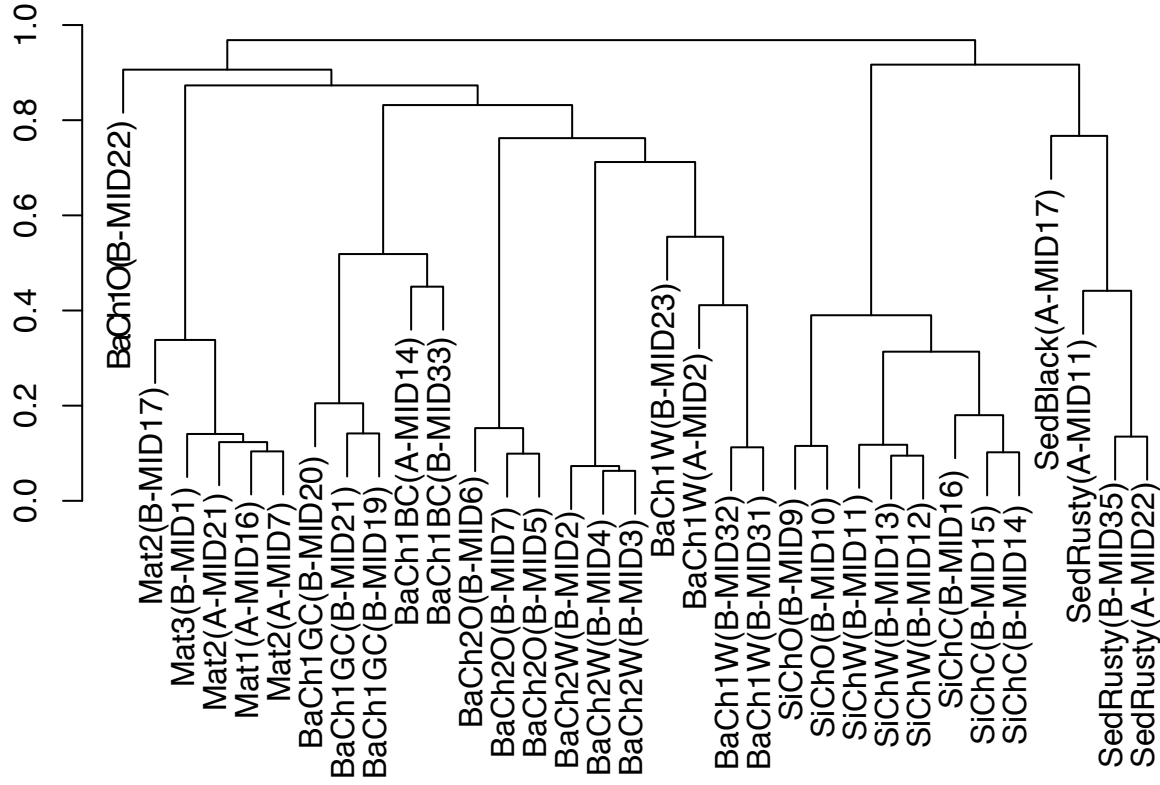

Supplement: Supplementary file 8 [file Image3.pdf]

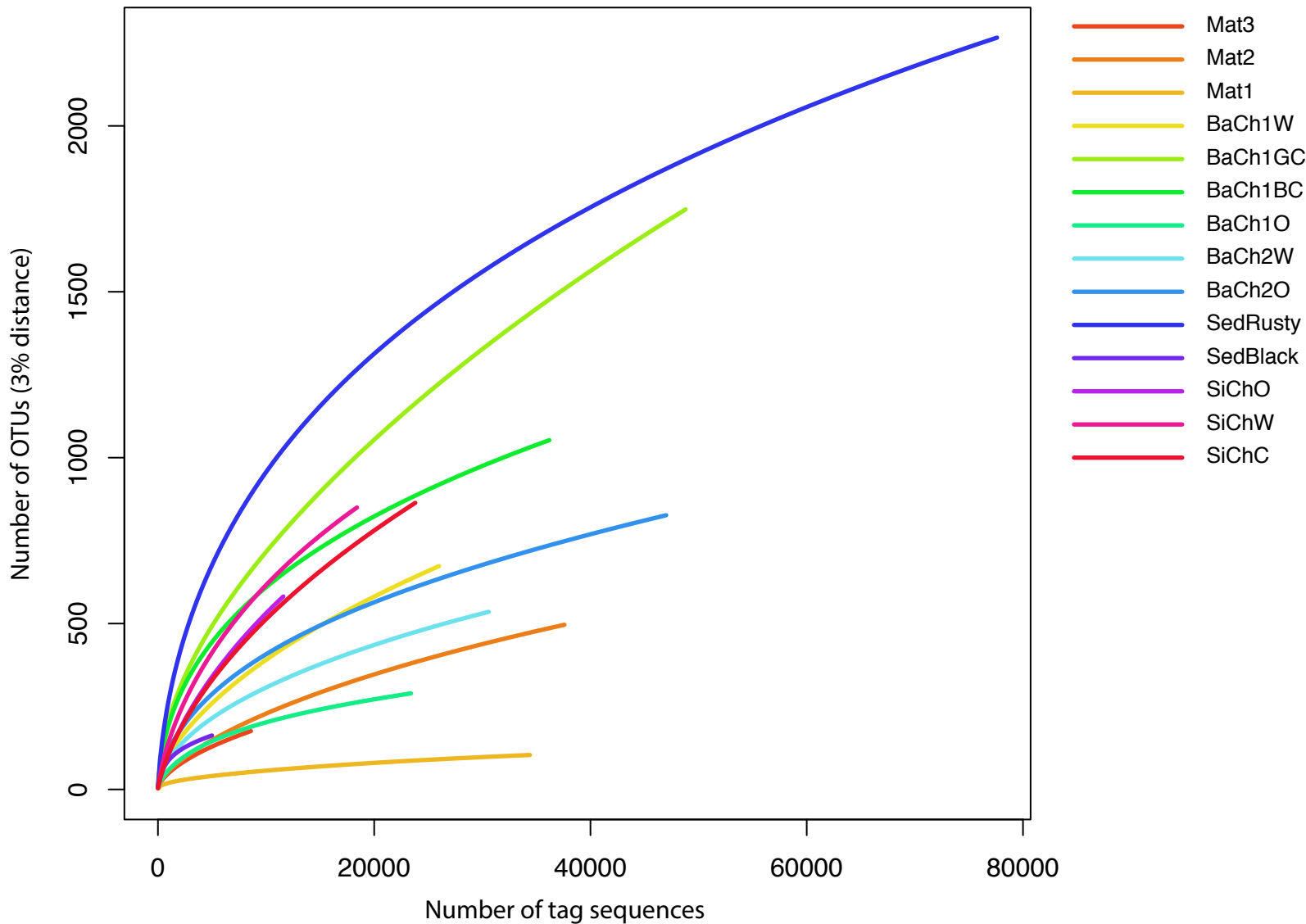

Supplement: Supplementary file 9 [file Image4.pdf]

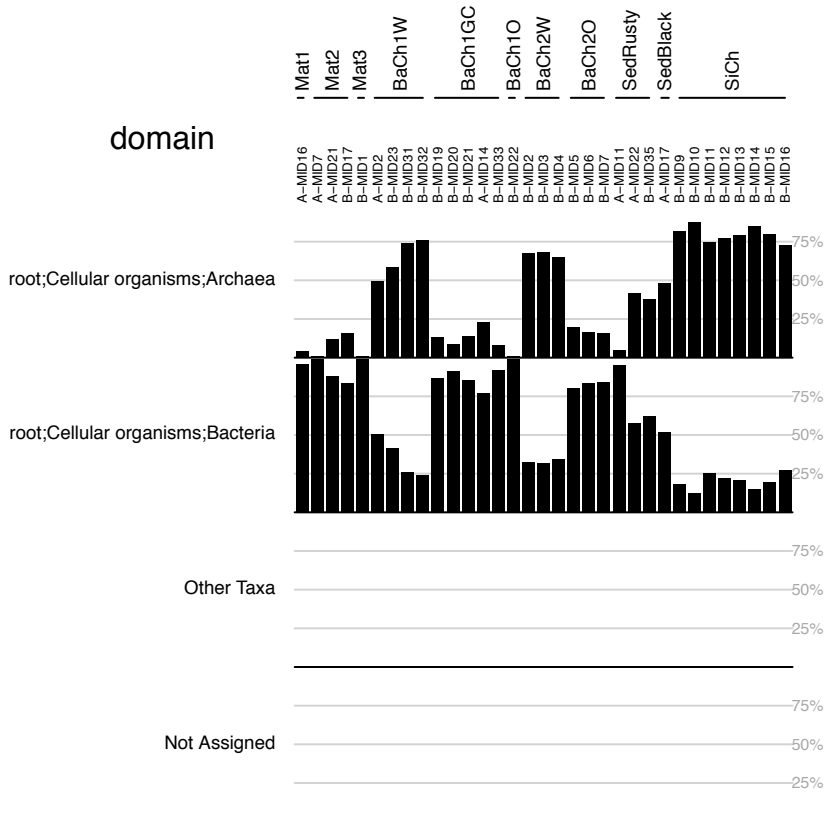

phylum

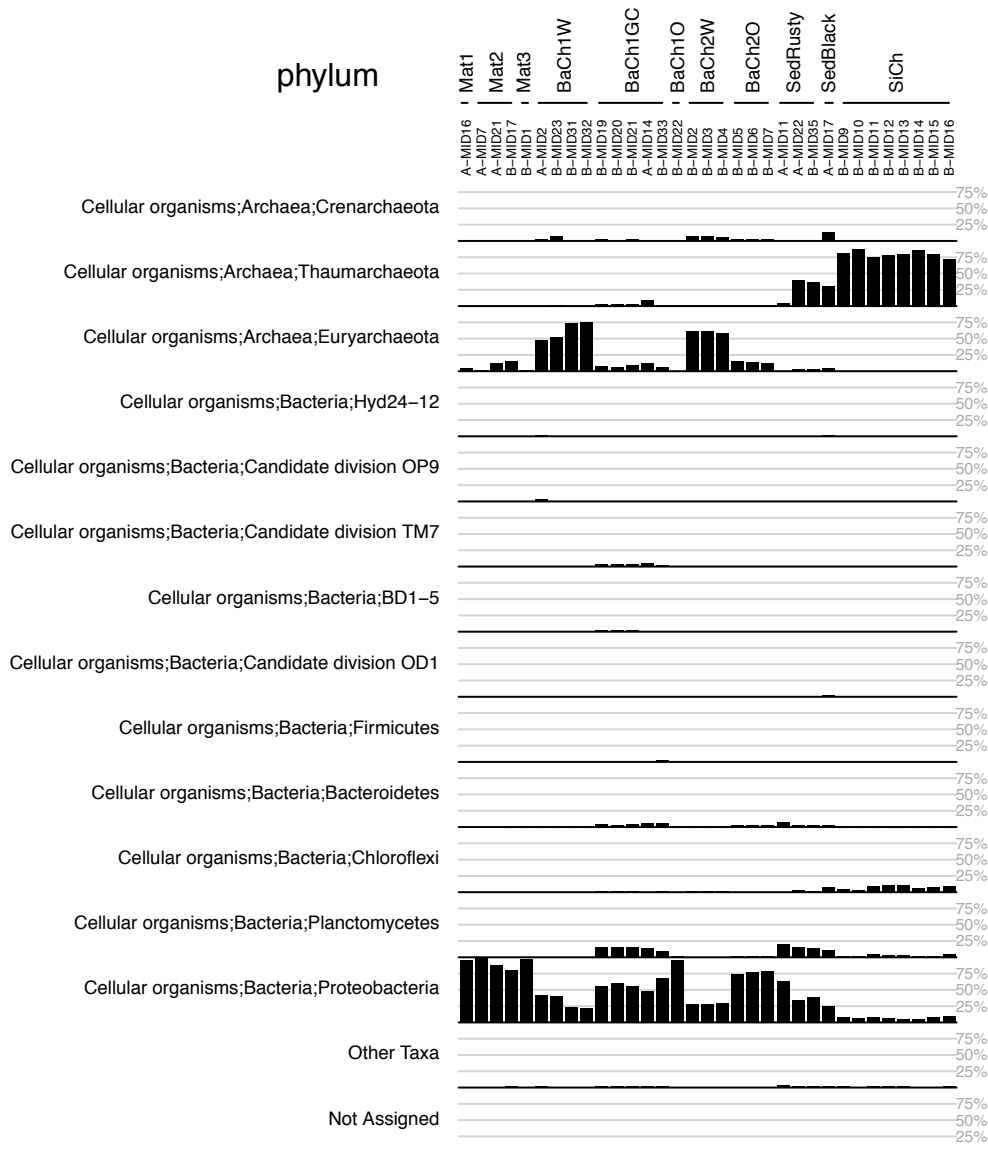

class

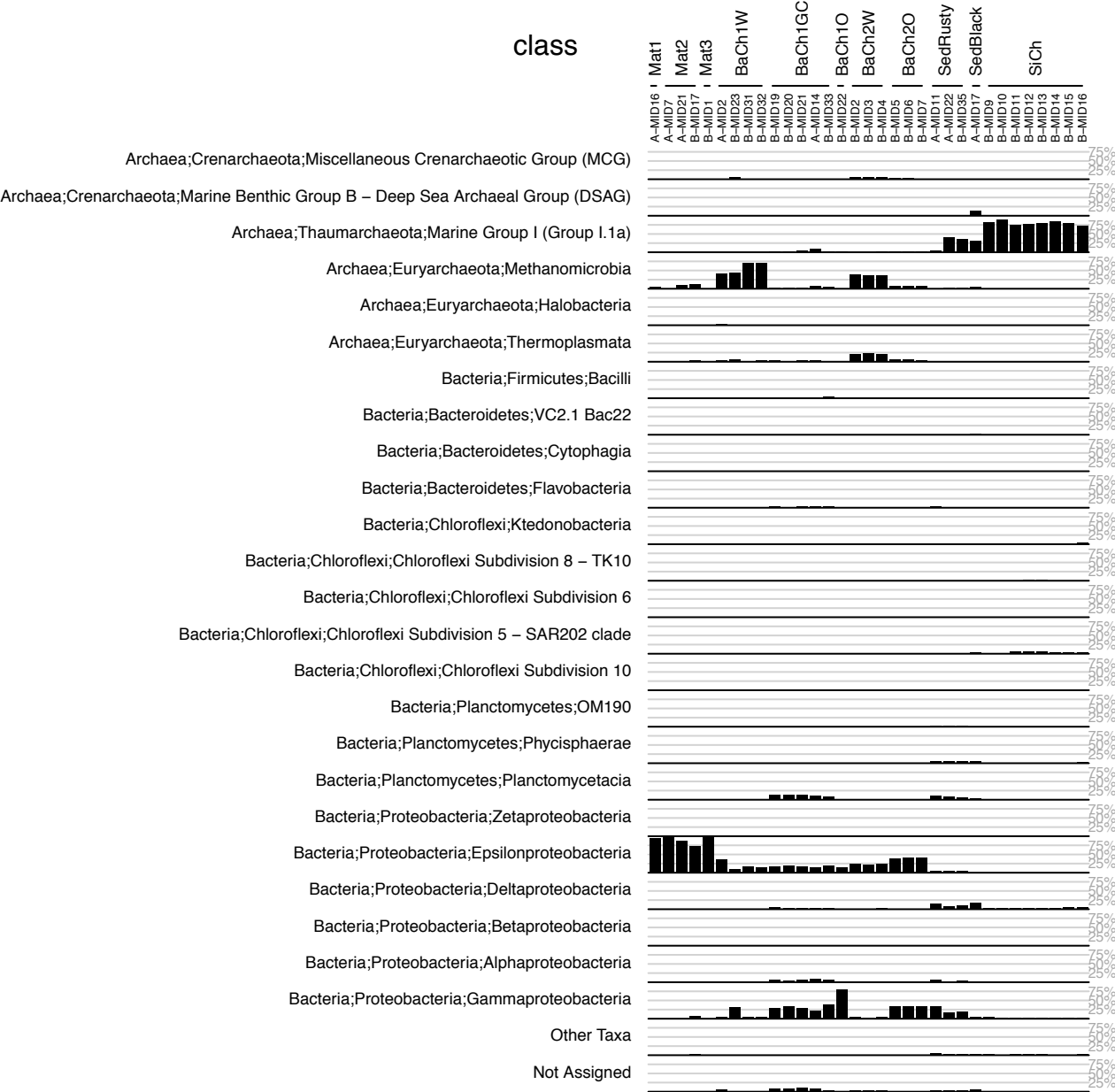

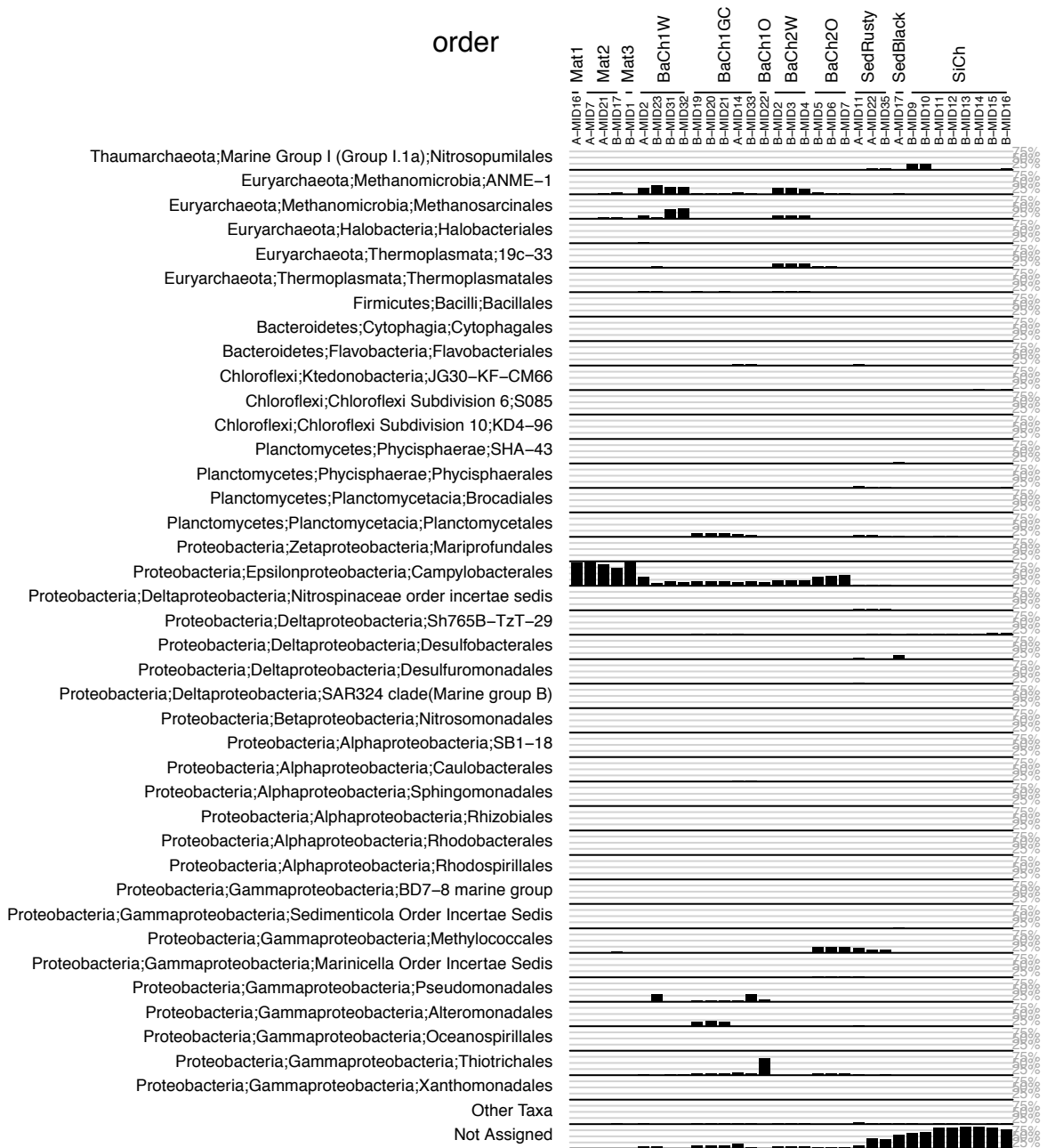

family

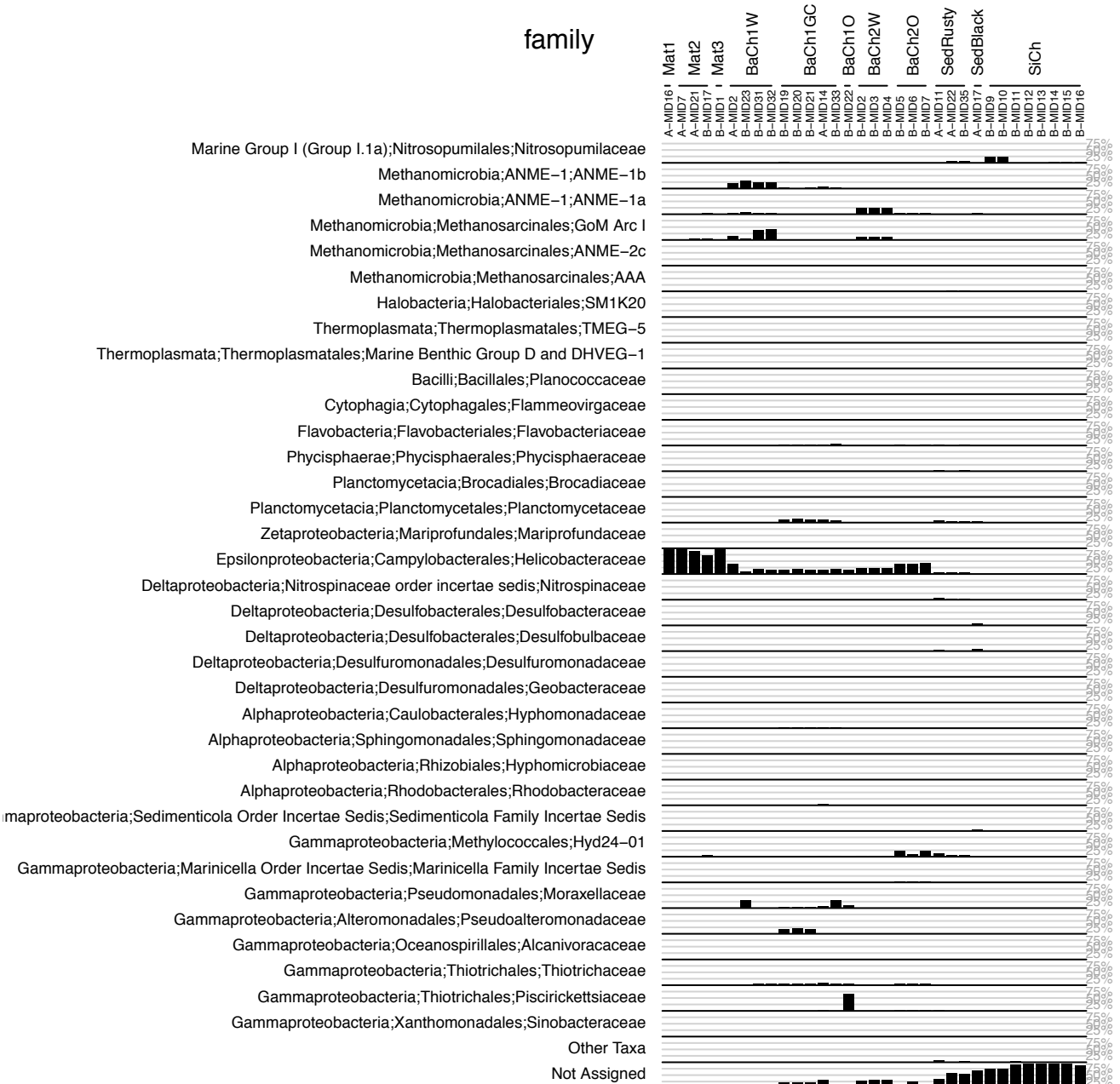

genus

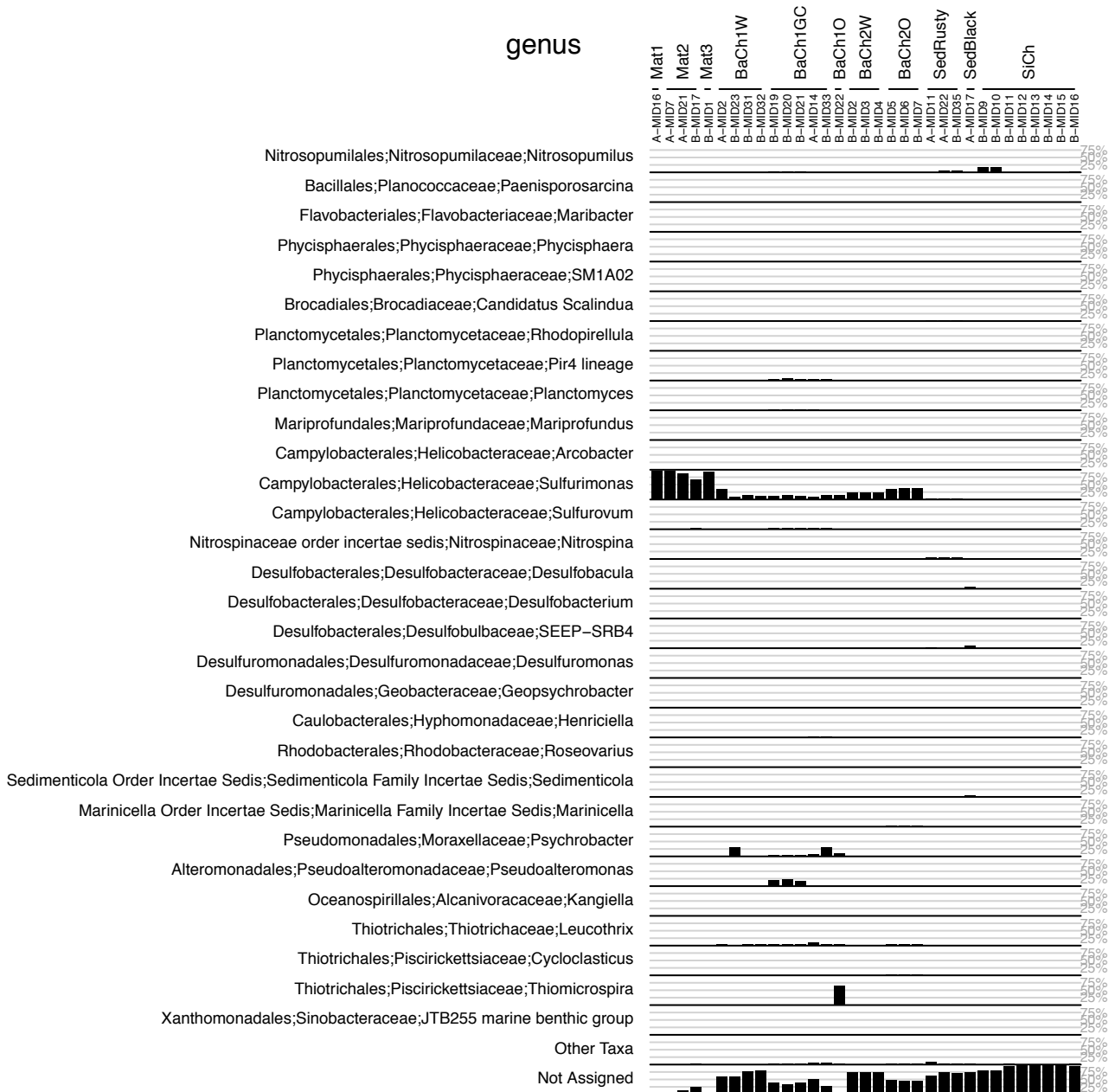

Supplement: Supplementary file 10 [file Image5.pdf]
